# The Role of Motor Learning in Spatial Adaptation near a Tool

**DOI:** 10.1371/journal.pone.0028999

**Published:** 2011-12-13

**Authors:** Liana E. Brown, Robert Doole, Nicole Malfait

**Affiliations:** 1 Department of Psychology, Trent University, Peterborough, Ontario, Canada; 2 Centre Nationale de la Recherche Scientifique, Institut de Neurosciences de la Timone (INT), Campus de Santé Timone, Marseille, France; University of Reading, United Kingdom

## Abstract

Some visual-tactile (bimodal) cells have visual receptive fields (vRFs) that overlap and extend moderately beyond the skin of the hand. Neurophysiological evidence suggests, however, that a vRF will grow to encompass a hand-held tool following active tool use but not after passive holding. Why does active tool use, and not passive holding, lead to spatial adaptation near a tool? We asked whether spatial adaptation could be the result of motor or visual experience with the tool, and we distinguished between these alternatives by isolating motor from visual experience with the tool. Participants learned to use a novel, weighted tool. The active training group received both motor and visual experience with the tool, the passive training group received visual experience with the tool, but no motor experience, and finally, a no-training control group received neither visual nor motor experience using the tool. After training, we used a cueing paradigm to measure how quickly participants detected targets, varying whether the tool was placed near or far from the target display. Only the active training group detected targets more quickly when the tool was placed near, rather than far, from the target display. This effect of tool location was not present for either the passive-training or control groups. These results suggest that motor learning influences how visual space around the tool is represented.

## Introduction

It is clear that one of the roles of multisensory neurons is to integrate visual, tactile, and proprioceptive information so that we can track where objects are located relative to our limbs, even when we are not looking at them directly. It may be this sensory integration, coupled with action-based predictions of upcoming sensory outcomes (‘efference copy’, [2], [3], that allows a skilled basketball player to quickly dribble and spin her way down the court while attending to the movements of her teammates and opponents.

Some bimodal visual-tactile neurons, discovered in the monkey ventral premotor cortex (PMv) and the intraparietal sulcus, have overlapping visual and tactile receptive fields (vRFs and tRFs, respectively), typically on the face or hand [4]–[9]. Some of these neurons also receive proprioceptive information [6]–[9] and their visual receptive fields are linked to hand motion such that the visual receptive field moves with the hand [6], [7]. Interestingly, the vRFs of these neurons surround and extend beyond the tRF such that visual stimuli appearing near but not touching the skin (within the vRF alone) can also recruit these neurons. Space near the hands and face is represented more densely than space far from the hands and face, and bimodal-cell firing rates gradually decay as the distance between the stimulus and the edge of the tactile RF increases [6], [7]. In short, visual information presented near the hands, i.e. in peripersonal or pericutaneous space [10], may recruit bimodal neurons, whereas visual information presented away from the hands may not recruit bimodal neurons.

Does this recruitment influence visual processing? In other words, do people treat visual information appearing near their hands differently? Although due to experimental limitations bimodal cells and their properties have not yet been documented in humans, reports of psychophysical experiments conducted both in neurological patients (e.g., [11]–[14]) and in groups of healthy participants ([15]–[17], but see [18]) suggest that people can detect visual targets more quickly and represent them more reliably when they appear near the hands. This difference in processing speed and reliability may arise from additional neurons recruited by targets appearing near skin of the hands relative to far from the hand. If the target falls within the visual RFs of bimodal cells, these cells may be recruited to help represent and process the target. In general, these benefits are not reliable when the patient or healthy participant sees a fake hand near the visual target [12], [15], [16]. This explanation for hand-proximity effects is reminiscent of the statistical facilitation that appears to explain redundancy effects, in which two identical stimuli are processed more quickly than one [19], [20]. Like redundancy effects, hand-proximity effects can be explained by the recruitment of additional neurons for processing, but for a single visual stimulus.

Like a basketball player, a skilled hockey player can weave the puck (the target) through opposing play with a stick (tool) while avoiding potential checkers. How is it that people are able to use tools to interact with objects almost as easily as they use their own hands? One part of the answer may be that, with experience, the multisensory integration associated with pericutaneous space extends beyond the hands to include a hand-held tool in its entirety. Iriki, Tanaka, & Iwamura [1] recorded from visual-tactile bimodal cells in the anterior bank of the intraparietal sulcus (a-IPS) both before and after their subjects (monkeys) practiced using a light, plastic rake to retrieve a food pellet. Before training, the visual receptive fields (vRF) of ‘distal’ cells – bimodal cells whose tRF is on the skin of the hand – surrounded the skin and space near the hands only, but after using the tool for five minutes, testing revealed that the same neurons now responded to stimuli presented at the tip of the tool. The conclusion was that the vRF adapted so that it included the space around the entire length of the tool. Likewise before training, the vRFs of ‘proximal’ cells – bimodal cells whose tRF was on the skin of the shoulder – encompassed the reach space of the arm and hand only, but after using the tool, the same vRFs grew to encompass the area reachable with the tool-in-hand. These changes were not induced by passive holding of the tool. The importance of training was underscored by Obayashi, Tanaka, & Iriki [21], who reported that hand-movement training caused previously unimodal somatosensory neurons in the post-central gyrus of the macaque parietal cortex [22] to become sensitive to near-hand visual stimuli (i.e. unimodal tactile neurons became bimodal neurons after training). In short, active use of the hand [21] or tool [1], [23] may change how bimodal cells represented the space surrounding the hand or tool.

Even though these neural properties have not been demonstrated in humans, experimental results from psychophysical studies conducted with human neurological patients and healthy participants indicate that tool use can change how nearby visual targets are processed ([12], [16], [24]–[32]; but see [33]). For example, researchers have demonstrated that near-space visual extinction extends to visual items appearing near the tip of a toy rake after the patient used the rake to retrieve distant objects [25], [31]. Berti and Frassinetti [24] showed that near-space hemispatial neglect expanded to far space when neglect patient PP held a stick-pointer but not when she held a laser-pointer. This latter result suggests that there may be a special role for objects whose reach (length) can be both seen and felt via tactile and proprioceptive cues signalling their inertia [34], [35].

The importance of active tool use (vs. passive holding) was demonstrated in a study conducted by Farnè et al. [25]. In a single patient with visual-tactile extinction, Farnè et al. showed that extinction extended to the tool tip after the patient used the tool to rake in objects for 5 minutes, but not after the patient spent that time passively holding the tool. In a follow-up study, Farnè, Iriki, and Làdavas [26] demonstrated that the strength of cross-modal extinction at the tip of the tool depended on the length of the tool used during training. Patients who trained with a 60 cm tool showed greater cross-modal extinction when holding a 60 cm tool than a 30 cm tool, and patients who trained with a 30 cm tool showed greater cross-modal extinction when holding the 30 cm tool. These results suggest that active training with the tool allows the user to learn about the capabilities of the tool from multiple sensory modalities.

Cardinali et al.[36] suggest that active tool training changes participants' implicit representation of the extent of their own limb, and report that after-effects of this adaptation temporarily change the way in which reaching movements are performed immediately after tool-use is discontinued. Therefore, adaptation following active tool use may change the way in which space around the tool is represented or it may change the way in which the limb is represented in the body. Ultimately, Cardinali et al. [10] argue that, at least with respect to tool-related spatial adaptation, there may be little difference between explanations couched in terms of peripersonal (or pericutaneous) space and a malleable body schema. On the whole, however, the following question remains unanswered.

**Figure 1:**
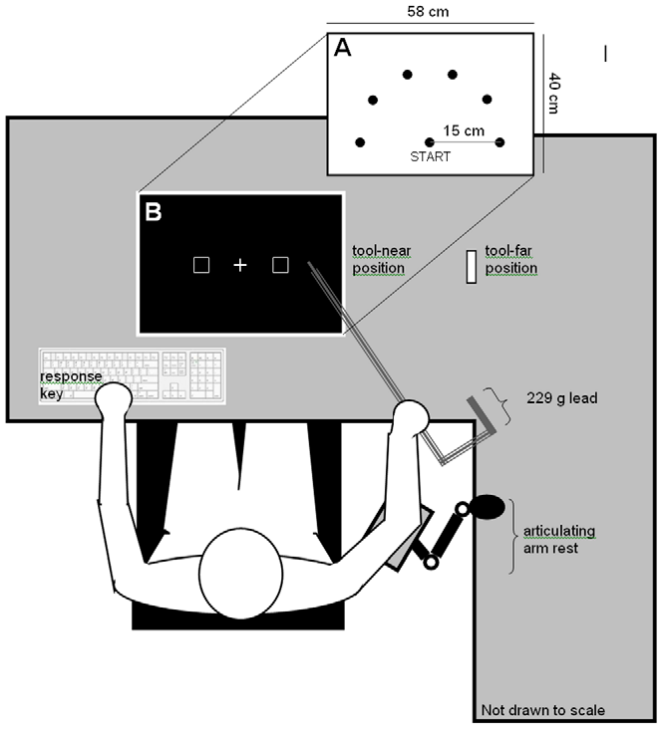
Experimental set up. The layout of the start position and targets for the motor learning task is shown in Panel A. The arrangement of the fixation cross and cue-target placeholders for the visual detection task is shown in Panel B.

Why does active tool use, and not passive holding, lead to spatial adaptation near a tool? One possibility is that tool-related spatial adaptation depends on motor adaptation: before a tool can be considered functional, the motor system must learn to predict and control the tool's inertia in response to forces applied both by gravity and the user. Another possibility is that tool-related spatial adaptation depends on visual adaptation: active tool use allows the user to see the length and spatial capability (reach) of the tool. We distinguished between these two hypotheses by employing a tool with novel dynamics to control for participants' prior history using pointing tools, and then isolating visual training with the tool from motor training. Participants made pointing movements to visible targets with the tool (see Figure 1A). Participants in the active training group performed self-generated actions; active training provided motor, visual, and kinesthetic experience with the new tool. Participants in the passive training group were moved passively to each target; passive training provided visual and kinesthetic experience with the tool, but no motor experience of how to wield the new tool. Finally, a no-training control group received no visual, kinesthetic or motor experience using the tool. After training, we measured how quickly participants detected visual targets using a cueing paradigm [16] in which we varied whether the tool was placed near or far from the target display (see Figure 1B). To preview, we found that tool-related spatial adaptation depends critically on motor learning: only the active training group responded more quickly to targets appearing near rather than far from the tool.

## Methods

### Participants

Sixty-eight right-handed undergraduate students from Trent University (51 women and 17 men, mean age±standard deviation  = 21.2±2.0 years, range 18–32) participated for extra credit or renumeration. All participants reported being right-handed [37], with normal or corrected-to-normal vision, and no history of any neurological or musculoskeletal disorder. The first 60 participants were pseudorandomly assigned to one of three (active, passive, and control) tool-training groups such that there was an equal number of participants in each group. The remaining eight participants were tested in a follow-up active-training experiment. The Trent University Research Ethics Board approved all procedures and each participant gave written informed consent before participation.

### Apparatus

For both the motor learning task and the visual detection task, participants sat at a table whose working surface (2011.6×91.4 cm) was 70 cm from the floor (see Figure 1). A portion of the table surface was removed and replaced with glass covered by translucent paper (58 cm×40 cm). A projector (Optoma DLP EP739, Optoma Technology, Inc, Mississauga, ON) and mirror were arranged below the table to allow the visual display to be projected up on to the glass surface. This region defined the participants' workspace. The chair was placed such that when the participant sat comfortably and extended her arms without leaning forward, her fingertips just touched the near edge of the workspace. Mean viewing distance from the bridge of the nose to the centre of this workspace was 104 cm. This seating arrangement ensured that the visual displays for both the motor learning and visual detection tasks were always beyond the normal (without tool) reach of the participants.

### Motor Learning Task

The tool was a 90 cm long hook-shaped plastic tube and it acted as a pointing device in the experiment (see Figure 1). The inertia of the tool was unpredictable from visual information as a 229 g mass (not visible to the participant) was added inside the tube to shift the location of its center of gravity lateral to the grasp location. Research shows that imposing a weight in this manner necessitates adaptation to bring the limb-tool system under control [38]–[41]. Participants were required to grip the tool so that the plane created by the hook and shaft of the tool was parallel to the work surface. A landmark on the tool was used to ensure that all participants grasped the tool at the same place. This requirement maximized the effect of the load on reaching movements made by the shoulder, elbow, and wrist. A Polhemus Liberty® (Polhemus, Burlington, VT) motion tracking stylus was secured to the tip of the pointer. Spatial position and orientation data, sampled at a rate of 100 Hz, were stored on a personal computer for later analysis.

Participants' right forearm were supported against gravity by an Ergorest® articulating armrest (Ergorest Oy, Siilinjärvi, Finland) while they performed reaching movements involving shoulder, elbow and wrist rotation. For the passive and the no-training control groups, the Ergorest® was used to help support the mass of the tool as well. Use of this device allowed participants in the passive condition to completely relax their arm as they were moved passively by the experimenter.

Six targets (2.0 cm in diameter) were presented in the horizontal table-top plane (see Figure 1A), arranged in a half-circle around a single start position (also 2.0 cm in diameter). All targets were 15 cm from the start position. The targets were labelled 1–6, beginning with the leftmost.

Three training conditions were contrasted. Training consisted of 138 movements to the six target locations, presented pseudorandomly so that each target location was presented 23 times. In the active training condition, participants used the tool to make a ballistic pointing movement from a single start location to one of six targets under their own volition. Participants were asked to point to the center of the target marker as quickly as possible. In the passive training condition, participants grasped the tool lightly but did not support its weight. The participant's arm was passively moved as the experimenter moved the tool tip to a target. Passive-training participants were asked to monitor the array and the tool's movements throughout the training phase and to let their arm rest completely in the arm-rest, so as to not interfere with the tool's motion. In the no-training control condition, participants held the tool passively without moving it for the same duration (15 minutes) as was required by participants in the active and passive groups to complete their training. The target array was visible during this time.

We assessed motor learning immediately following training by asking all participants to perform a six-trial pointing transfer task in which participants used the tool to actively point once to each target in a randomly-presented order. For the active training group, the transfer task was identical to the training task, and for the passive and no-training groups, the transfer task was their first and only exposure to the tool's unusual inertial properties. The transfer task was limited to six trials to prevent adaptation to these inertial properties by the passive and no-training groups.

### Visual Detection Task

Immediately following the transfer test, all participants completed the following visual detection task. The visual detection task consisted of a modified version of Posner's cueing task [16], [42]. As mentioned above, the display was projected up onto the work surface from below. This arrangement allowed the participants to view the detection items on same horizontal workspace in which they had previously trained with the tool. All display items were presented on a black background. At the beginning of each trial a fixation cross (1.4°×1.4°) was presented at body midline at a mean viewing distance of 104 cm flanked by two white-bordered square location placeholders (1.8°×1.5°) located 5.5° (10 cm) on either side of fixation (see Figure 1B). After a variable foreperiod (500–1500 ms) one of the placeholders brightened for 200 ms and followed immediately by the presentation of a circular target (1.4° diameter) either in the centre of the brightened placeholder (validly cued target) or in the other placeholder (invalidly cued target). The target remained on-screen until the participant responded or until the display timed out at 1200 ms. On 12% of trials, no target was presented; participants were instructed to avoid responding on these trials. These catch trials were included to encourage participants to attend to the display. The display was programmed using the Psychophysics Toolbox extensions [43], [44] for Matlab (The Mathworks™, Natick, MA).

The visual detection task used a 2 – cue location (left, right) by 2 – target location (left, right) by 2 – tool proximity (near, far from the display) within-subjects design. Cue location varied pseudorandomly such that there were an equal number of right and left cues. The target was also presented an equal number of times on the left and right, but it was tied to cue location such that on 68% of trials, the target appeared in the cued location (valid cue). On 20% of trials, the target appeared in the uncued location (invalid cue), and on 12% of trials, no target was presented. Tool proximity to the display was blocked. On half of the blocks, participants held the tool such that the tip laid next to (within 5 cm of) the right target placeholder, and on half the blocks, participants held the tool such that the tip laid 30 cm to the right of the right target (see Figure 1). Participants responded to the presentation of the target by pressing the keyboard space bar with their left hand. Each participant completed 6 blocks of 50 trials. We measured response latency (reaction time; RT in ms) and percent correct.

### Data Analysis

Both training and transfer-test movements were analysed. Analysis programs written with Matlab were used to define the beginning and end of each pointing movement. The onset and the end of the movement were defined as the time when the resultant velocity of the tool-tip first exceeded and then first fell below 20 mm/s for five consecutive samples, respectively. Secondary corrective movements were excluded from the analysis. We measured movement time (MT), signed end-point error along the azimuth (horizontal dimension) and in depth, and end-point variability. For the training phase, end-point variability was computed in successive and exclusive bins of 23 trials (mean radial error from the mean end-point for each participant within each bin). Both movement time and end-point variability were submitted to a mixed 2-group by 6-trial bin analysis of variance (ANOVA; α = .05). For the transfer phase, MT, signed error, and end-point variability (mean radial error from target location for each participant) were submitted to a one-way ANOVA with training group as the factor.

We submitted the visual detection task RT data to a 3 – group (active, passive, control) x 2 – cue location (left, right) x 2 – target location (left, right) x 2 – tool proximity (near, far) mixed ANOVA, where group was the between-subjects factor and cue location, target location, and tool proximity were within-subjects factors. We restricted our RT analyses to correct responses. Response accuracy (percent correct) data were submitted to a similar ANOVA, and measures of detection sensitivity (d') and response bias (β) were computed within each condition ([45], Table 1). Significant interactions were decomposed by conducting simple effects analyses. Main effects involving group were further investigated using planned comparisons (t-tests).

**Table 1:** Measures of performance accuracy (percent correct), sensitivity (d'), and response bias (β) in the visual detection task.

Training Condition	Tool Position	TargetLocation	% Correct (95% CI)	Hits (%)	Misses (%)	CorrectReject (%)	FalsePositive (%)	d'(sensitivity)	β (response bias)
Control	Far	Left	99.1 (1.6)	88.0	0	11.1	.89	4.5	0.024
		Right	99.0 (1.5)	88.0	0	11.0	.95	4.5	0.023
	Near	Left	98.7 (1.9)	88.0	0	10.8	1.21	4.4	0.019
		Right	98.9 (1.7)	88.0	0	10.9	1.03	4.5	0.021
Passive	Far	Left	98.5 (1.6)	88.0	0	10.6	1.50	4.2	0.016
		Right	99.4 (1.5)	88.0	0	11.1	.61	4.7	0.032
	Near	Left	98.7 (1.8)	88.0	0	10.8	1.27	4.3	0.018
		Right	99.1 (1.6)	88.0	0	11.1	.85	4.6	0.025
Active	Far	Left	98.7 (1.7)	88.0	0	10.7	1.26	4.3	0.018
		Right	98.6 (1.6)	88.0	0	10.7	1.33	4.3	0.018
	Near	Left	98.5 (2.0)	88.0	0	10.7	1.47	4.3	0.016
		Right	98.5 (1.7)	88.0	0	10.5	1.54	4.2	0.016

## Results

### The active training group adapted to the inertia of the novel tool

**Figure 2:**
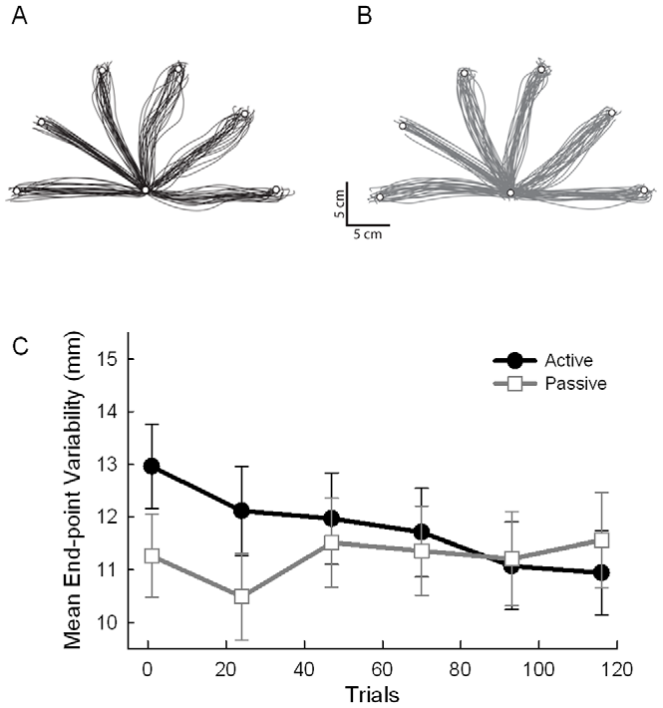
Motor learning results. Training trajectories from a representative participant in the active (participant JD) and passive training groups (participant LP) are shown in Panels A and B, respectively. Panel C shows mean end-point variability of the training movements for the active and passive training groups over trial bins of 23 trials. Error bars represent 95% confidence intervals.

Figure 2 shows all training movement-path trajectories from two representative participants from the active (Panel A) and passive (Panel B) training groups. The group by trial-bin ANOVA revealed a significant interaction of group and trial bin, *F*(1, 38) = 5.41, *p* = .025. The active training group showed a significant reduction in variability with practice, *F*(5, 90) = 5.81, *p* <.001, indicating that early in training the additional load affected the active group's ability to control the tool (see Figure 2C). In contrast and as expected, kinematic variability remained constant throughout the trials for the passive training group (*p* = .742).

This effect of motor learning for the active group was confirmed by the transfer test that immediately followed the training phase. Movement time (MT), signed end-point error along the azimuth and in depth (from the participant's perspective), and end-point variability were submitted to separate one-way ANOVAs with training group (active, passive, no training) as the sole factor.

**Figure 3:**
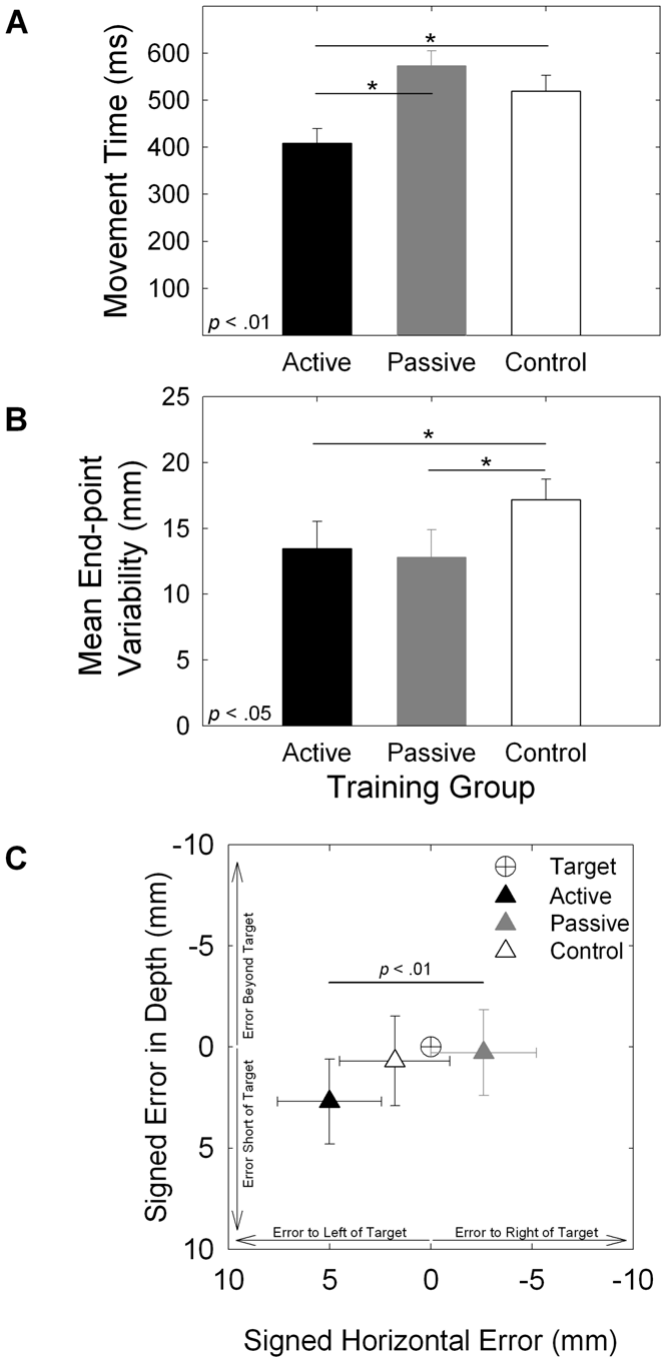
Motor learning results for the test phase. Panel A: Movement time (ms) as a function of training group, Panel B: mean end-point variability (mm) as a function of training group, Panel C: mean signed error (mm) as a function of training group. Error bars represent 95% confidence intervals.

The analysis of movement time revealed a significant main effect of group, *F*(2, 133) = 10.013, *p* <.001 (see Figure 3A). Planned comparisons showed that the active training group (*M* = 349 ms, *SEM* = 20 ms) had shorter MTs than both the passive training group, (*M* = 476 ms, *SEM* = 19 ms; *p* <.001), and the no-training control group (*M* = 451 ms, *SEM* = 20 ms; *p* <.001).

The analysis of end-point variability also revealed a significant main effect of group, *F*(2, 133) = 3.566, *p* = .031 (see Figure 3B). Although pointing errors for the active (*M* = 13.9 mm, *SEM* = 1.5 mm) and passive (*M* = 13.2 mm, *SEM* = 1.4 mm) groups did not differ (*p* = .734), both of these groups were less variable than the control group (*M* = 18.3 mm, *SEM* = 1.5 mm), *p* = .037, and *p* = .014, respectively.

Analyses of signed end-point error along the azimuth and in depth revealed a significant difference between training groups along the azimuth only, F(2, 321) = 8.36, p <.001. Whereas the active group tended to miss the target by landing to the right of the target, the passive group tended to land to the left of the target (Figure 3C). Neither the active- nor passive-training groups were significantly different from the no-training group. There was no effect of training group on signed end-point error in depth, F(2, 321) = 1.45, p = .236.

Together, these findings demonstrate that (1) altering the tool's inertia by adding a load influenced participants' ability to control the tool, and (2) participants with active training were able to move the tool significantly faster than the other training groups while maintaining the same or better levels of precision. In other words, the active training group was able to control the tool better than the other groups.

### Tool location influenced visual detection latency in the active training group

The degree to which the participants were able to detect the target accurately, measured by percent correct, is high averaging 98% overall (see Table 1). We submitted the arcsin transform of percent correct rates to a 3 – training group x 2 – tool location x 2 – target location mixed analysis of variance. This analysis revealed a main effect for target location, F(1, 59) = 4.31, p = .042. Participants were significantly more accurate when the target appeared on the right (M = 98.9%, SEM = .2%) than on the left (M = 98.6, SEM = .2%). Critically, there was no group x hand position interaction, F(2, 59) = .11, p = .899, and no three-way interaction between group, hand position, and target location, F(2, 59) = .46, p = .634. Measures of sensitivity are also presented in Table 1. There is no notable change in either sensitivity (d') or response bias (β) as the experimental conditions change.

**Figure 4:**
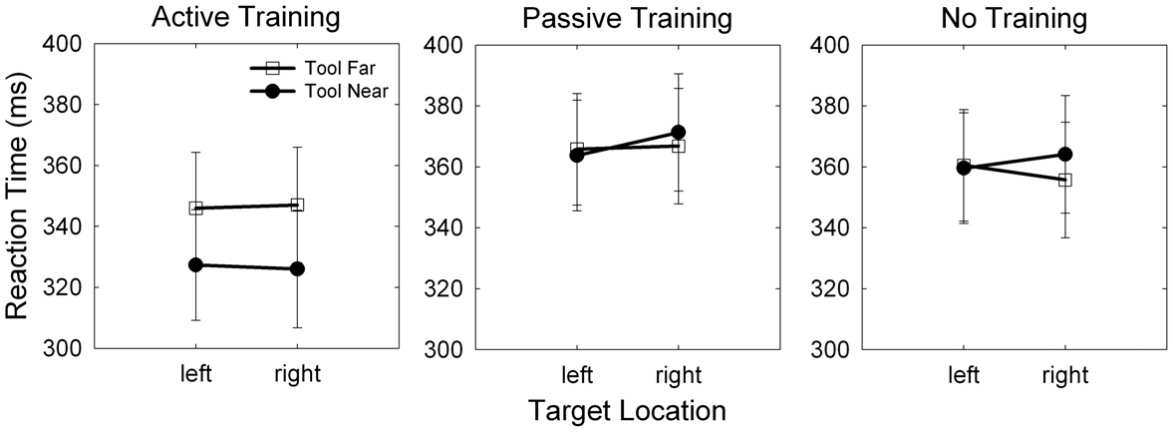
Reaction time (RT; ms) as a function of training condition, tool location, and target location. The active training group responded to targets more quickly when the tool was held near the display rather than far from it (training condition x tool location interaction: p = .014). This effect did not interact with target location (3-way interaction, p = .114). Error bars represent 95% confidence intervals.

Does visual processing time depend on tool location and training condition? To answer this question we asked participants to respond as quickly as possible to a target that could appear either to the left or right of fixation (target location) with the tool placed either near or far from the right target (tool proximity). We measured reaction time (RT; ms) and submitted it to a mixed ANOVA. Training condition was the between-subjects factor and cue location, target location, and tool proximity were within-subjects factors. The analysis revealed a significant interaction between training condition and tool proximity, *F*(2, 57) = 4.57, *p* = .014 (see Figure 4). Simple effects analysis showed that there was a significant effect of tool proximity for the active group, for whom the tool-near condition (M = 327 ms, SEM = 9 ms) was significantly faster than the tool-far condition (M = 346 ms, SEM = 9 ms), *F*(1, 19) = 8.26, *p* = .010, Cohen's d (corrected for dependence between means using Morris and DeShon's equation 8 [46]) = .64. There was no effect of tool-proximity for the passive (*p* = .840) or no-training (*p* = .482) groups. We also found a significant main effect of training condition, *F*(2, 57) = 3.36, *p* = 0.042. Participants in the active-training group (*M* = 337 ms, *SEM* = 9 ms) responded significantly more quickly than participants in the passive-training group (*M* = 367 ms, *SEM* = 9 ms; *p* = .016) and the no-training group (*M* = 360 ms, *SEM* = 9 ms; *p* = .048).

We expected that tool position would interact with target location such that when the tool was placed near the target display, participants would respond faster to targets appearing on the right side of the display (5 cm from the tool tip) in comparison to targets appearing on the left side of the display (25 cm from the tool tip). The three-way interaction of training condition, target location and tool proximity was not significant, *F*(2, 57) = 2.25, *p* = .114. This means that the improvement in target detection time when the tool was placed near the display was present for targets that appeared both to the left and right of fixation.

Electrophysiological recordings in monkeys have shown at least two general types of arm-related bimodal neurons [1], [6]. Neurons with tactile RFs on the distal aspect of the limbs (the hands) have corresponding visual RFs that are limited in extent to the space near the hands. By contrast, bimodal cells with tactile RFs on the more proximal aspect of the upper limb (the shoulder) have corresponding visual RFs that appear to include much of the spatial range of motion of the upper limb [1], [23]. We considered the possibility that, because we used a multi-segment reaching task that involved rotations mostly at the shoulder and elbow, motor learning engaged and induced spatial adaptation in multisensory systems linked to the shoulder. To test whether the extent of spatial adaptation depends on whether motor training involves proximal or distal musculature, we recruited eight new participants for a new active training condition that involved reaching with primarily wrist and elbow musculature. In this condition, participants' elbows were fixed on the table-top and they grasped the tool using a power grip; they wielded the tool using only their wrist and elbow. This arrangement effectively eliminated the overt contribution of shoulder joint rotations to the reaching action during training. The training and test procedures for this new active wrist-training group were identical to those for the former active (shoulder) training group. We submitted the visual task RT data from the wrist-training group to a two-way target location (left, right) by tool proximity (near, far) repeated measures ANOVA and found a significant main effect of tool location. Targets were processed significantly more quickly when the tool was placed near the display (M = 369, SEM = 4 ms) than when the tool was placed far from the display (M = 380, SEM = 4 ms), *F*(1, 7) = 5.68, *p* = .049. There was no main effect of target location, *F*(1, 7) = .13, *p* = .726, and there was no interaction of tool proximity and target location, *F*(1, 7) = .30, *p* = .599. Even after restricting movement training to the elbow and wrist, placing the tool near the display benefited both the left and right target locations.

The placement of the responding left hand on the left side of the display may have invoked faster responding to targets appearing on the left than on the right due to the compatibility of the stimulus location and the response location (Simon effect; [47]). Importantly, because the tool proximity effect was predicted to invoke faster responding to targets appearing on the right than on the left, it is possible that the Simon effect masked the presence of the tool-proximity effect. To address this possibility, we checked for the Simon effect by analyzing the RT data from the tool-far condition only. They were submitted to a 3–training condition by 2–target location mixed ANOVA. This analysis revealed that none of the training groups responded more quickly to targets appearing on the left side of the display (on the same side of fixation as the responding hand) than on the right, *F*s <1. Likewise, in the wrist-training group, the interaction between tool location and target location was not significant (p = .599): even when the tool was placed far from the display, responses to left-side targets (M = 379, SEM = 5 ms) were not significantly faster than responses to right-side targets (M = 382, SEM = 5 ms; p = .323). Therefore it is unlikely that compatibility effects influenced the outcome of this study.

### Visuospatial orienting to a visual cue was not influenced by training condition or tool location

**Figure 5:**
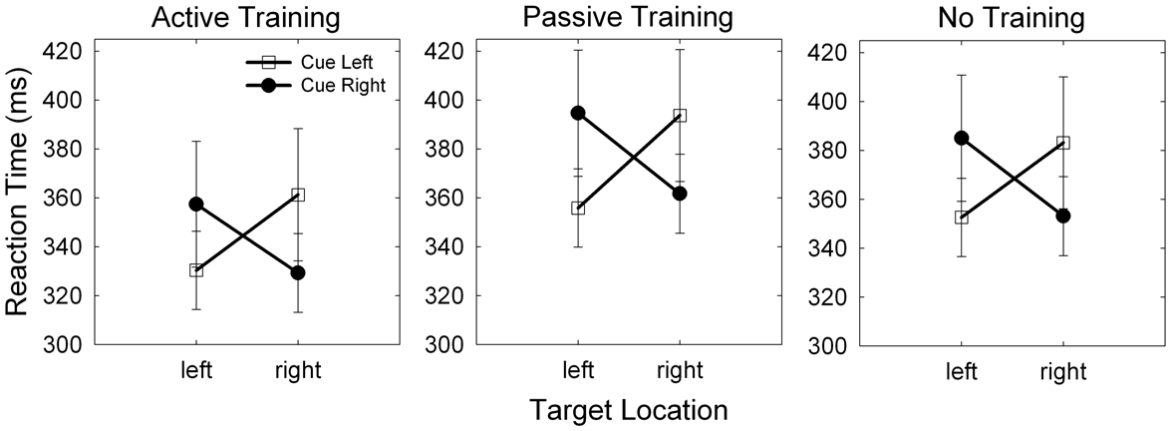
Reaction time (RT; ms) as a function of cue location, target location and training condition. RT was lower when the target appeared in the cued location than in the uncued location, p <.001 [61]. This effect did not interact with training condition (p = .861) or tool location (p = .145). Error bars represent 95% confidence intervals.

Across all groups, but not to a larger extent in any particular one, we found reduced RTs when the cue accurately predicted target location *F*(1, 57) = 49.64, *p* <.001 [42]. When shown the left cue, participants responded more quickly to a target that appeared on the left (*M* = 346 ms, *SEM* = 5 ms) than on the right (*M* = 379 ms, *SEM* = 8 ms). Similarly, when shown the right cue, participants responded more quickly to targets that appeared on the right (*M* = 379 ms, *SEM* = 7 ms) than on the left (*M* = 348 ms, *SEM* = 5 ms; Figure 5). This effect did not interact with training condition, *F*(2, 57) = .15, *p* = .861, or tool proximity, *F*(1, 57) = 2.18, *p* = .145, and the four-way interaction was not significant, *F*(2, 57) = .637, *p* = .533. The lack of interaction between training group, tool proximity and cueing effect is one indicator that the mechanism responsible for the facilitation of target detection in the tool-near condition is independent of the mechanism responsible for exogenous orienting of visual attention to externally cued locations [47].

## Discussion

Neurophysiological evidence suggests that the visual receptive field (vRF) of visual-tactile bimodal neurons can grow to encompass a hand-held tool, but that this spatial adaptation follows active tool use, not passive holding [1]. In humans, studies suggest that tool related spatial adaptation exists (17,24–27,30,31,36 but see 33) and depends on active tool use [26], [29], [32]. Here we addressed possible reasons for this dependence on active use in humans. Tool-related spatial adaptation may depend on motor adaptation: before a tool can be functionally linked to spatial information, the motor system must learn to predict and control the tool's inertia in response to forces applied both by gravity and the user. By contrast, tool-related spatial adaptation may depend on visual adaptation: before a tool can be linked functionally to spatial information, active tool use allows the user to see the length and spatial capability (reach) of the tool. We distinguished between these two hypotheses by employing a tool with novel dynamics and then isolating visual training with the tool from motor training. First, we found that people who received active tool training could control the tool better than people who received passive (experimenter-guided) or no training with the tool: participants in the active training group could point to targets with the tool quickly and precisely. In a post-training test of target detection speed, we found that only participants who trained actively with the tool responded more quickly to targets when the tool tip was placed near rather than far from the display area. The speeded response may arise from additional neural power for targets appearing near the tip of the tool relative to targets appearing far from the tip. If the target falls near the tip of the tool, within the adapted visual RF of bimodal cells, these cells will be recruited to help process the target, speeding its detection and the eventual response. This explanation for near-tool effects is not unlike the statistical facilitation that appears to explain redundancy effects, in which two identical stimuli are processed more quickly than one because of the additional neural recruitment that is invoked by the second stimulus (e.g., [19], [20]). The results converge with others demonstrating that active tool use changes the way that visual stimuli on or near the tool are processed ([12], [17], [24]–[27], [29]–[32], but see [33]), and they suggest that this change is driven not by visual experience with the tool alone, but by motor learning.

### Tool-related benefits may depend on motor learning

We found that near-tool benefits were measurable when visual experience with the tool was paired with motor experience. Near-tool effects were absent in our passive training condition, however, even though participants did observe the tool move to different regions of space. This result might shed a different light on other findings that apparently stand in contrast to it. For example, Holmes et al. [29] reported a reduction in interference associated with near-tool visual stimuli after only a very short duration of active tool use, and Maravita et al. [32] found cross-modal interference for near-tool visual stimuli after simple tool holding (a condition akin to our control condition), even though it was – without active training – restricted to the visual field in which the hand was placed. Our result seems to indicate that the impact of visual experience alone on tool-related spatial adaptation was observed in these studies because participants used tools that were very easy to wield or that they may have learned to manipulate previously, like pointing sticks and toy garden or sport tools. Here, we controlled for participants' motor-skill history by asking them to wield a stick-like tool with novel inertial properties.

Our results indicate that at least in the case in which one is using a novel tool with unknown inertial properties, motor learning may play an important role in tool-related spatial adaptation. Motor learning involves establishing a reliable predictive relationship between the planned motor command and the visual, proprioceptive, and dynamic tactile sensory consequences resulting from its execution [49]–[51]. Tool motor learning includes acquiring the ability to predict the sensory information that will result from both limb and tool movement. According to one account, an inverse model of the limb is used to transform planned movement trajectory information into a motor command: the precisely-timed muscle contractions that are required to propel the hand or tool to the reaching target. A forward model of the reaching movement is used to predict the sensory outcomes of that motor command. The inverse model must account for many factors, including physical factors like the mass and lengths of limb segments, gravity, and both directly- and indirectly-generated (interaction) torques about the joints [39], [40], [51], [52]. When additional masses, like tools, are added to the limb or hand, both the forward and inverse model must adapt to account for this additional mass [38], [39], [41]. If participants have worked with the tool before, this adaptation may be expedited as they access previously-stored information about the tool's inertial profile [53]. This motor adaptation allows the user to make predictions about the spatial location of the working end of the tool as it is moved, linking limb, hand, and tool posture (signalled by the somatosensory system) to locations in space beyond the body (usually signalled by the visual system). The primary claim forwarded here is that when one is presented with an unfamiliar tool with unknown inertial properties, tool-related spatial adaptation, perhaps resulting from the adaptation of the vRF of visual-tactile bimodal neurons, depends on the establishment of a reliable internal model of the tool. Put differently, we may need to be able to control and reliably predict the tool's actions before changes in which the space around the tool is represented can be implemented.

This proposal is consistent with our current findings and with the findings of Farnè, Iriki, and Làdavas [26], who demonstrated that the strength of spatial adaptation at the tip of the tool depended on the length or inertial properties of the tool used during training [34], [35]. When patients trained with a 60 cm tool, cross-modal extinction was greater with a 60 cm tool than a 30 cm tool, and vice versa. The proposal is also consistent with the position of bimodal neurons within the parietal and premotor cortices, brain regions associated with visuomotor processing and motor planning. Visual-tactile bimodal neurons, whose visual receptive fields grow to incorporate tools, are well positioned to receive information about the reliability of the motor command and of the predicted sensory outcome [1], [6].

### Factors influencing the extent of tool-related spatial adaptation

The pattern of the tool-proximity effect that we observed suggests that it may depend on the region of space in which training occurred. We took care to ensure that the region of space where training targets were presented overlapped the region of space where visual detection targets were presented. When the tool was placed within the region of training, we found a general benefit for visual detection targets presented there. By contrast, when the tool was placed outside the region of training, responses to visual detection targets were significantly slower, even though both targets were still clearly within reach of the tool. This result is consistent with findings from the motor-learning literature which suggest that adaptation to novel dynamics is spatially localized [54]. If motor learning has limited generalization beyond the trained space, and if tool-related spatial adaptation depends on motor learning, then it follows that tool-related spatial adaptation, as indexed by speeded detection of targets presented near the tool tip, should depend on whether both the tool and the targets are presented in the trained space.

We also found that placing the tool near the display benefited targets presented both to the right and left of fixation, both near and relatively far from the tool-tip, respectively. The results of the follow-up experiment indicate that this effect did not depend on whether motor training primarily involved rotations at the shoulder and elbow or at the elbow and wrist. Many stereotyped actions, like reaching, take advantage of available synergies (e.g., [55]–[57]). Indeed, Debicki & Gribble [58]–[59] have shown that even when the shoulder joint is stabilized by an exoskeleton, shoulder muscular activation generated in response to single-joint elbow reaching movements remains unchanged in comparison to when the shoulder is not stabilized. In other words, it is likely that when people are asked to learn to use a relatively large tool with novel dynamics, this always invokes muscular contributions from the shoulder, regardless of the degree of overt rotation at the shoulder. Likewise, it is possible that when people are asked to learn to use a relatively large tool with novel dynamics, tool-related spatial adaptation may be invoked both in proximal and distal multisensory neurons [1] and therefore benefits may not be confined to the space very near the tool tip.

### Can tool-related benefits be explained by spatial attention?

Another possible explanation for the effects of tool-proximity on target detection time observed here is that the tool-tip simply draws exogenous spatial attention to the area near the tool and that perhaps the effectiveness of exogenous spatial attention also depends on one's ability to reliably predict the tool's motion. The data we present, however, suggest that exogenous attention cannot be the sole mechanism driving near-tool effects. We found robust cueing effects [42]: people responded more quickly when the target appeared in the cued location than in the uncued location. This effect is thought to index the shift of exogenous spatial attention in response to the appearance of both the cue and the target. According to additive-factors logic [48], two experimental factors (in this case, cue location and tool location) that engage the same cognitive process (in this case, exogenous spatial attention) should interact with one another and experimental factors that engage different cognitive or neural processes should not interact with one another. We found that neither tool location nor training condition influenced the cueing effect, supporting the notion that the processes responsible for tool-proximity effects are independent of the processes responsible for the orienting of exogenous spatial attention in this experiment. This finding does not rule out, however, the possibility that participants allocate endogenous attention to the tool location [33], [60], assuming that the allocation of endogenous attention is sensitive to motor learning.

### Conclusion

How is it that people are able to use tools to interact with objects as easily as they use their own hands? One part of the answer may be that multisensory integration extends beyond the hands to include hand-held tools. Our results suggest that motor learning – learning that results in the establishment of a predictive relationship between commands generated by the motor system and the visual, tactile, and proprioceptive consequences of both limb and tool movement – may play a role in this process.
